# Combined transrectal ultrasound and radiomics model for evaluating the therapeutic effects of neoadjuvant chemoradiotherapy in locally advanced rectal cancer

**DOI:** 10.1007/s00384-024-04792-8

**Published:** 2025-01-07

**Authors:** Dilimire Abuliezi, Yufen She, Zhongfan Liao, Yuan Luo, Yin Yang, Qin Huang, Anqi Tao, Hua Zhuang

**Affiliations:** 1 Department of Medical Ultrasound, West China Hospital of Sichuan University, 37# Guo Xue Xiang, Chengdu, 610041 Sichuan China; 2 Department of Gastroenterology and Hepatology, West China Hospital of Sichuan University, 37#Guo Xue Xiang, Chengdu, 610041 Sichuan China

**Keywords:** Transrectal ultrasound (TRUS), Radiomics, Locally advanced rectal cancer (LARC), Neoadjuvant chemoradiotherapy (NCRT), Tumor regression grade (TRG)

## Abstract

**Purpose:**

This study aimed to explore a combined transrectal ultrasound (TRUS) and radiomics model for predicting tumor regression grade (TRG) after neoadjuvant chemoradiotherapy (NCRT) in patients with locally advanced rectal cancer (LARC).

**Methods:**

Among 190 patients with LARC, 53 belonged to GRG and 137 to PRG. Eight TRUS parameters were identified as statistically significant (*P* < 0.05) for distinguishing between the groups, including PSV_pre_, LD_post_, TD_post_, CEUS-IG_post_, LD change rate, TD change rate, RI change rate, and CEUS-IG downgrade. The accuracies of these individual parameters in predicting TRG were 0.42, 0.62, 0.56, 0.68, 0.67, 0.70, 0.63, and 0.71, respectively. The AUC values were 0.596, 0.597, 0.630, 0.752, 0.686, 0.660, 0.650, and 0.666, respectively. The multi-parameter ultrasonic logistic regression (MPU-LR) model achieved an accuracy of 0.816 and an AUC of 0.851 (95% CI: [0.792–0.909]). The optimal pre- and post-treatment radiomics models were RF (Mean-PCA-RFE-6) and AE (Zscore-PCA-RFE-12), with accuracies of 0.563 and 0.596 and AUCs of 0.601 (95% CI: [0.561–0.641]) and 0.662 (95% CI: [0.630–0.694]), respectively. The combined model (US-RAD_pre_-RAD_post_) showed the highest predictive power with accuracy and AUC of 0.863 and 0.913.

**Conclusions:**

The combined model based on TRUS and radiomics demonstrated remarkable predictive capability for TRG after NCRT. It serves as a precision tool for assessing NCRT response in patients with LARC, impacting treatment strategies.

**Supplementary information:**

The online version contains supplementary material available at 10.1007/s00384-024-04792-8.

## Introduction

Colorectal cancer is the third most common cancer globally and the second leading cause of cancer-related deaths. Recently, the incidence and mortality rates of colorectal cancer have increased, particularly in younger age groups [1]. Locally advanced rectal cancer (LARC) accounts for approximately 50% of all rectal cancer cases and is defined as clinical stage II (T3-4N0M0) or III (T1-4N1-2M0). The standard treatment for LARC is neoadjuvant chemoradiotherapy (NCRT), followed by total mesorectal excision (TME). After NCRT, tumors exhibit various histological and morphological changes, which are termed tumor regression. These changes, such as cellular necrosis, fibrotic tissue proliferation, inflammatory cell accumulation, foamy cell degeneration, and calcification, are used to assess NCRT effectiveness using the tumor regression grade (TRG), which can currently be graded only by postoperative pathology [2]. Studies suggest that accurate preoperative prediction of TRG, allowing for a “watch-and-wait” approach in patients achieving pathological complete response (pCR), can improve survival quality by avoiding surgical complications and permanent colostomies, thus facilitating personalized treatment and precision medicine [3].

Imaging examinations play a crucial role in predicting the TRG. MRI T2WI has been used to observe the proportion of residual tumor and fibrosis post-NCRT, proposing magnetic resonance tumor regression grade (mrTRG); however, its accuracy is debatable [4, 5]. Recently, transrectal ultrasound (TRUS) has become an important tool for evaluating and managing LARC and assessing tumor characteristics such as size, location, and infiltration depth to predict TRG and guide clinical decisions [6]. Radiomics, a novel image analysis technique, introduced by Lambin in 2012, overcomes the subjectivity of conventional image interpretation by quantitatively analyzing and identifying subtle features in images that are difficult to detect with the naked eye [7]. It has been increasingly applied to predict the therapeutic effect of NCRT in patients with LARC; however, the findings are not consistent and its applicability needs further validation [8–10]. Currently, few studies have applied ultrasound-based radiomics to predict TRG. This study, therefore, aimed to evaluate the value of a combined TRUS and radiomics model to predict TRG after NCRT in patients with LARC.

## Materials and methods

### *Patient selection*

The inclusion criteria were as follows: (1) biopsy-confirmed solitary rectal cancer, (2) preoperative NCRT, (3) TRUS examinations before and after NCRT, (4) TME surgery with complete pathological results, and (5) complete clinical and pathological data. The exclusion criteria were as follows: (1) intestinal stenosis-limiting TRUS examination, (2) distant metastasis or other malignancies, (3) ultrasound post-NCRT interval of > 10 weeks [11], and (4) poor ultrasound image quality. TRG grading followed the 7th edition of the American Joint Committee on Cancer (AJCC) [12].

Between January 2017 and December 2022, a total of 478 patients with LARC were recorded. After rigorous screening based on the inclusion and exclusion criteria, ultimately 190 patients were included in the study (43 cases did not receive NCRT; 26 cases had intestinal stenosis; 78 cases had distant metastasis; 39 cases had an interval of more than 10 weeks between TRUS after NCRT; 30 cases did not undergo surgery or were lost to follow-up; 72 cases had poor ultrasound image quality). To minimize selection bias, the above data were entered using a double-entry method, where two individuals independently entered the same data, which were then compared. The objective was to achieve a power of 80% with an alpha level of 0.05. Based on preliminary assumptions, the required sample size was calculated to be 126. Consequently, the sample size of 190 patients surpassed the minimum requirement, thereby ensuring the statistical reliability of the study. This study was conducted and reported in accordance with the STROBE guidelines.

### Image collection and ultrasound acquisition

This study used a Biosound MyLab Twice ultrasound diagnostic system (Italy) with a TRT33 probe (dual-plane transrectal probe, convex array emission frequency 3–9 MHz, linear array emission frequency 4–13 MHz) and a BK Medical BK5000 (Denmark) with an E14CL4b probe (dual-plane transrectal probe, probe frequency range 14–4 MHz). TRUS examination was performed by two gastroenterological ultrasound physicians with more than 7 years of experience.

**Fig. 1:**
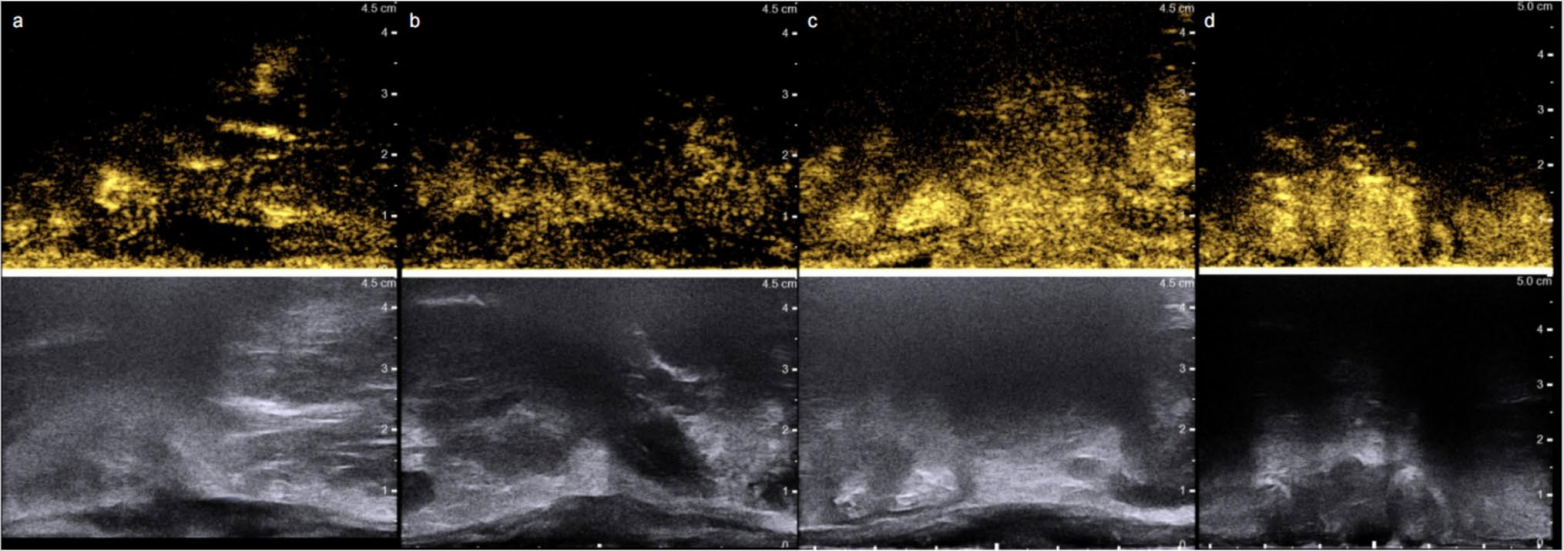
**a** CEUS-IG Grade 1: Lesions exhibit non-uniform enhancement with extremely uneven distribution, potentially including large non-enhancing areas. **b** CEUS-IG Grade 2: Lesions display suboptimal enhancement and may present middle areas with hypo-enhancement. **c** CEUS-IG Grade 3: Lesions show relatively uniform enhancement with the possibility of layered enhancement or small patchy hypo-enhanced areas. **d** CEUS-IG Grade 4: Lesions demonstrate uniform enhancement, with little to no variation in enhancement degree or distribution

Prior to examination, the patients were told to empty their bowel contents. The patients were asked to assume a left-lateral position, following which a probe was inserted with a coupling agent to reduce air gaps. The length diameter (LD) and thickness diameter (TD) were measured on the largest longitudinal section of the tumor, followed by color blood flow collection in the CDFI mode. The peak systolic velocity (PSV) and resistive index (RI) were measured in the main artery of the tumor. After 2.4 ml of sulfur hexafluoride microbubble contrast agent (Bracco, Italy) was injected into the median cubital vein, the contrast videos were stored for approximately 1 min. Contrast-enhanced ultrasound inhomogeneity grade (CEUS-IG) was based on peak contrast images. The CEUS-IG grading criteria [13] are illustrated in Fig. 1. Pre- and post-NCRT ultrasound parameters included change rate = (pre-treatment—post-treatment)/pre-treatment × 100%; CEUS-IG downgrade was assessed based on pre- and post-NCRT grading. The image selection criteria were as follows: (1) clear, artifact-free 2D ultrasound images and (2) removal of highly repetitive images per case.

### Ultrasound radiomics workflow

#### Tumor delineation and feature extraction

**Fig. 2:**
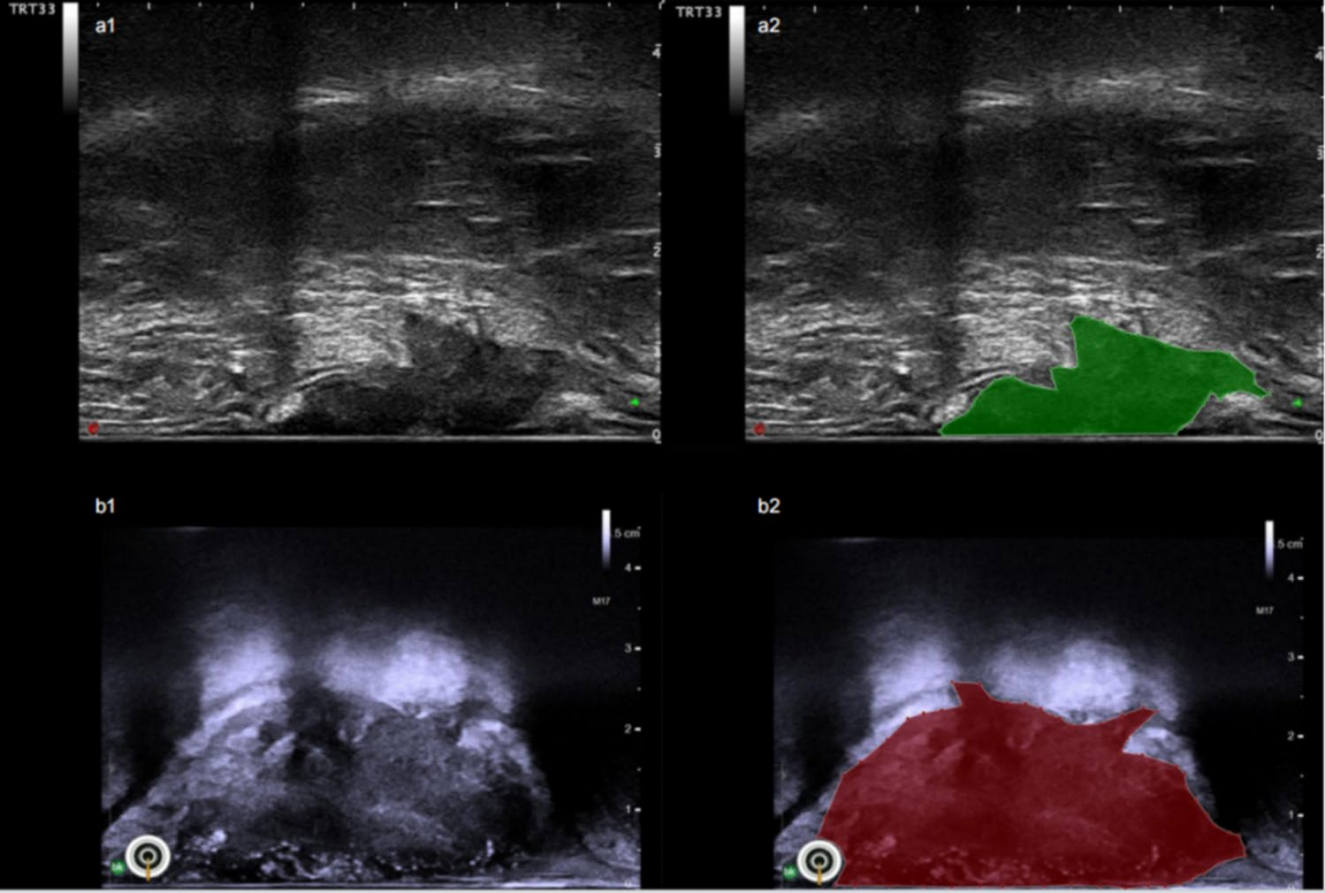
Delineation of the LARC lesions in the TRUS images. (a1-a2) Acquired by Biosound MyLab Twice ultrasound diagnostic system before NCRT, the TRUS image (a1) and mask image (a2) of the lesion (TRG 1). (b1-b2) Acquired by BK Medical BK5000 ultrasound diagnostic system after NCRT, the TRUS image (b1) and mask image (b2) of the lesion (TRG 3)

Two-dimensional TRUS images were imported to the Labelme workstation (github.com/wkentaro/labelme, version 5.1.1). A physician with 3 years of gastroenterological ultrasound experience outlined the tumor areas in the ultrasound images, blinded to patient details (Fig. 2). For consistency in image segmentation, another physician with 7 years of experience supervised and reviewed the process. Feature extraction utilized Pyradiomic [14], an open-source Python package for medical imaging. The extracted features included first-order statistics, shape, gray-level co-occurrence matrix (GLCM), gray-level run length matrix (GLRLM), gray-level size zone matrix (GLZM), neighboring gray tone difference matrix (NGTDM), and gray-level dependence matrix (GLDM) applied to the original and wavelet-transformed image types.

#### Feature normalization, dimension reduction, selection, and model building

Feature normalization utilized the mean and Z-score methods to eliminate inconsistencies across different measures. Dimension reduction employed principal component analysis (PCA) and Pearson correlation coefficients (PCC) to simplify data complexity and prevent overfitting. Feature selection involved multiple methods, including analysis of variance (ANOVA), recursive feature elimination (RFE), the relief algorithm, and the Kruskal–Wallis (KW) test (detailed descriptions of these methods provided in the supplementary material 1). After feature selection, different normalization methods, dimension types, and feature selection approaches were used to build seven radiomics models: support vector machine (SVM), autoencoder (AE), linear discriminant analysis (LDA), random forest (RF), logistic regression (LR), LR-Lasso, and Gaussian process (GP). Tenfold cross-validation of the training set was used to assess model fit and robustness. Models were evaluated using the area under the curve (AUC) on the cross-validation set. Model analysis and building were performed using the FeAture Explorer (https://github.com/salan668/FAE) in Python (https://www.python.org/) [15].

### Statistical methods

This study employed SPSS 26.0, R 4.3.2, and the FeAture Explorer software. Quantitative data are expressed as mean ± standard deviation or median (IQR) and compared using an independent *t*-test or Mann–Whitney *U* test. Qualitative data are presented as frequencies or rates and compared using the chi-square test. A multi-parameter ultrasound regression model was constructed, and fit was assessed using the Hosmer–Lemeshow test. ROC curves were used to evaluate the diagnostic performance of the different models. AUC, accuracy, sensitivity, specificity, positive predictive value (PPV), and negative predictive value (NPV) were calculated to evaluate the performance of the different models. Normo diagrams and calibration curves were drawn for the combined model. The Delong test and clinical decision curve analysis (DCA) were used to compare the efficacy of the different models. *P* < 0.05 was considered to be statistically significant.

## Results

### Demographic and TRUS data

This study included 190 patients. Based on the AJCC pathological TRG grading standards, 53 patients were divided into a good response group (GRG), and 137 patients were divided into a poor response group (PRG). A total of 4083 pre- and post-treatment TRUS images from patients with LARC were collected, of which 3128 were selected after screening. The images were randomly divided into training and test sets in a 7:3 ratio for both pre- and post-treatment. The patient characteristics and TRUS image distributions are summarized in Table 1.

**Table 1:** General characteristics of enrolled patients

	GRG	PRG	Total	*P* value^*^
*N*	53	137	190	
TRG (*N*)	TRG 0 (23)	TRG 2 (62)		
	TRG 1 (30)	TRG 3 (75)		
Age (mean ± SD)	57.36 ± 10.66	58.30 ± 10.14		.900
Gender (*N*: male, female)	(40, 13)	(108, 29)		.697
Number of images (pre-NCRT, post-NCRT)	917 (415, 502)	2211 (831, 1380)	3128	

*GRG* good response group, *PRG* poor response group, *compared with use of *t*/*χ*^2^ test, *P* < 0.05

### Multi-parameter ultrasonic logistic regression (MPU-LR) model

#### Comparison of TRUS parameters between GRG and PRG

As shown in Tables 2 and 3, statistically significant differences were observed between the two groups for the following parameters: pre-NCRT PSV, post-NCRT LD, post-NCRT TD, post-NCRT CEUS-IG, LD change rate, TD change rate, RI change rate, and CEUS-IG downgrade (*P* < 0.05).

**Table 2:** Comparison of TRUS parameters between good response group and poor response group

Group	LD_pre_ (mm)	TD_pre_ (mm)	PSV_pre_ (cm/s)	RI_pre_	LD_post_ (mm)	TD_post_ (mm)	PSV_post_ (cm/s)	RI_post_
GRG	39 (20.5)	15 (7.5)	11.6 (8.9)	0.78 (0.16)	25 (14)	9 (3)	9.2 (6.18)	0.66 (0.15)
PRG	37 (15)	14 (7)	12.5 (10.05)	0.75 (0.17)	30 (16)	10 (6)	11.2 (7.4)	0.71 (0.16)
*Z* value	− 1.423	− 0.879	− 2.058	− 1.838	− 2.079	− 2.791	− 1.855	− 1.882
*P* value*	0.155	0.379	0.04*	0.066	0.038*	0.005*	0.064	0.06
Group	LD change rate (%)		TD change rate (%)		PSV change rate (%)		RI change rate (%)	
GRG	0.35 (0.21)		0.38 (0.36)		0.16 (1.08)		0.14 (0.24)	
PRG	0.17 (0.36)		0.21 (0.37)		0.15 (0.81)		0.03 (0.22)	
*Z* value	− 3.979		− 3.42		− 0.243		− 3.202	
*P* value*	0.000*		0.001*		0.808		0.001*	

*TRUS* transrectal ultrasound, *GRG* good response group, *PRG* poor response group, *LD* longitudinal diameter, *TD* thickness diameter, *PSV* peak systolic velocity, *RI* resistive index, *compared with the Mann–Whitney *U* test, *P* < 0.05

**Table 3:** Comparison of CEUS-IG and CEUS-IG downgrade between GRG and PRG

Group	CEUS-IG_pre_				CEUS-IG_post_			
	1 Grade	2 Grade	3 Grade	4 Grade	1 Grade	2 Grade	3 Grade	4 Grade
GRG	14	17	11	11	23	23	7	0
PRG	27	30	53	27	26	27	64	20
*χ*^2^ value	6.066				36.204			
*P* value	0.108				0.000*			
Group	CEUS-IG downgrade							
	0 Grade (not downgraded)				1 Grade (downgraded)			
GRG	23				30			
PRG	105				34			
*χ*^2^ value	17.732							
*P* value	0.001*							

*TRUS* transrectal ultrasound, *GRG* good response group, *PRG* poor response group, CEUS-IG contrast-enhanced ultrasound inhomogeneity grade, *compared with use of *χ*^2^ test, *P* < 0.05

#### Predictive efficiency of single TRUS parameters and its cutoff values

Eight TRUS parameters with differences between the two groups were used to predict NCRT response, as shown in Table 4. The cutoff value for the pre-NCRT PSV was 22 cm/s, with an AUC of 0.596. The cutoff value for post-NCRT LD was 28 mm, with an AUC of 0.597. The cutoff value for post-NCRT TD was 11 mm, with an AUC of 0.630. The cutoff value for post-NCRT CEUS-IG was grade 3, with an AUC of 0.752. The cutoff value for the LD change rate was 26%, with an AUC of 0.686. The cutoff value for the TD change rate was 38%, with an AUC of 0.660. The cutoff value for the RI change rate was 8.00%, with an AUC of 0.650. The cutoff value for the CEUS-IG downgrade was grade 1, with an AUC of 0.666.

**Table 4:** Prediction efficiency and cutoff values of TRUS parameters

Parameters	Cutoff values (units)	Accuracy	Sensitivity	Specificity	PPV	NPV	AUC
PSV_pre_	22 (cm/s)	0.42	0.98	0.20	0.04	0.96	0.596
LD_post_	28 (mm)	0.62	0.66	0.61	0.18	0.82	0.597
TD_psot_	11 (mm)	0.56	0.77	0.47	0.16	0.84	0.630
CEUS-IG_post_	3 Grade	0.68	0.87	0.61	0.08	0.92	0.752
LD change rate	26 (%)	0.67	0.75	0.64	0.55	0.45	0.686
TD change rate	38 (%)	0.70	0.55	0.76	0.53	0.47	0.660
RI change rate	8 (%)	0.63	0.7	0.61	0.59	0.41	0.650
CEUS-IG downgrade	1 Grade	0.71	0.57	0.77	0.52	0.48	0.666

*TRUS* transrectal ultrasound, *PPV* positive predictive value, *NPV* negative predictive value, *AUC* area under the curve, *LD* longitudinal diameter, *TD* thickness diameter, *PSV* peak systolic velocity, *RI* resistive index, *CEUS-IG* contrast-enhanced ultrasound inhomogeneity grade

#### MPU-LR model

**Fig. 3:**
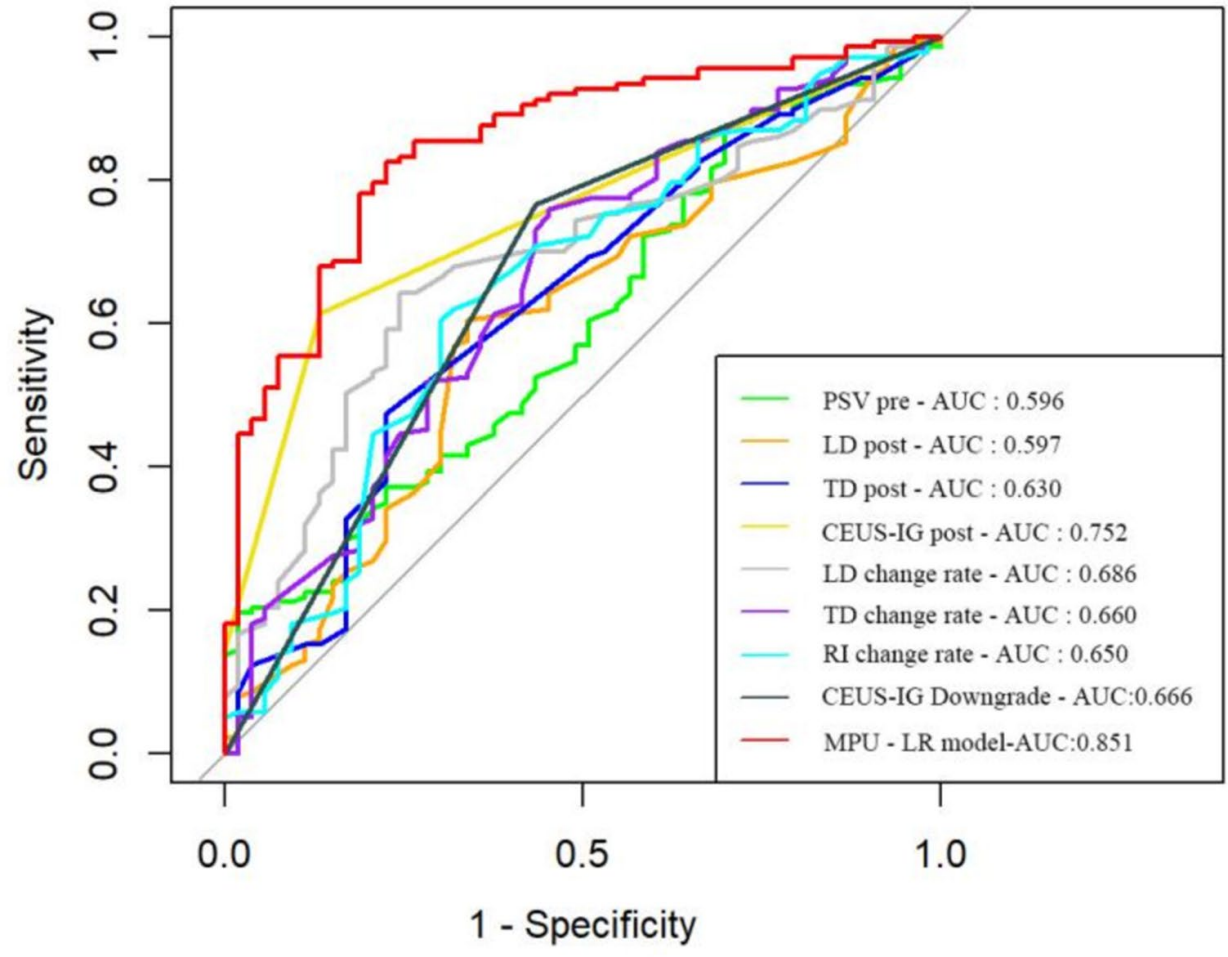
ROCs and AUCs of MPU-LR model and conventional ultrasound parameters

The eight ultrasound parameters with intergroup differences (PSV_pre_, LD_post_, TD_post_, CEUS-IG_post_, LD change rate, TD change rate, RI change rate, and CEUS-IG downgrade) were included in the logistic regression analysis. PSV_pre_, CEUS-IG_post_, LD change rate, and RI change rate were identified as independent factors influencing the TRG (*P* < 0.05) (Table 5). The MPU-LR model showed an accuracy of 0.816, sensitivity of 0.566, specificity of 0.912, PPV of 0.845, and NPV of 0.71, with an AUC of 0.851 (95% CI: [0.792–0.909]). The Hosmer–Lemeshow test indicated a good fit for the model (*χ*^2^ = 5.88, *P* = 0.661). Moreover, the predictive efficacy of the MPU-LR model was significantly higher than that of a single ultrasound parameter (Fig. 3).

**Table 5:** Results of multi-parameter TRUS logistic regression analysis

Parameters	Estimate	Standard error	*Z* value	*P* value	*OR* value	*OR* value (95% CI)
PSV_pre_	0.067	0.032	2.094	0.036	1.070	[1.009, 1.145]
LD_post_	0.014	0.020	0.680	0.496	1.014	[0.976, 1.058]
TD_post_	− 0.027	0.053	− 0.499	0.618	0.974	[0.874, 1.081]
CEUS-IG_post_	0.972	0.247	3.929	< 0.001	2.643	[1.655, 4.390]
LD change rate	− 2.543	1.041	− 2.442	0.015	0.079	[0.009, 0.547]
TD change rate	− 0.954	0.860	− 1.109	0.267	0.385	[0.066, 1.927]
RI change rate	− 2.093	1.039	− 2.014	0.044	0.123	[0.014, 0.851]
CEUS-IG downgrade	− 0.724	0.423	− 1.714	0.087	0.485	[0.211, 1.112]
Constant	− 0.762	1.019	− 0.748	0.454	0.467	[0.063, 3.503]

*PSV* peak systolic velocity, *RI* resistive index, *CEUS-IG* contrast-enhanced ultrasound inhomogeneity grade, *OR* odds ratio; *P < 0.05

### Ultrasound radiomics

#### Feature extraction, normalization, dimension reduction, selection, and modeling

**Fig. 4:**
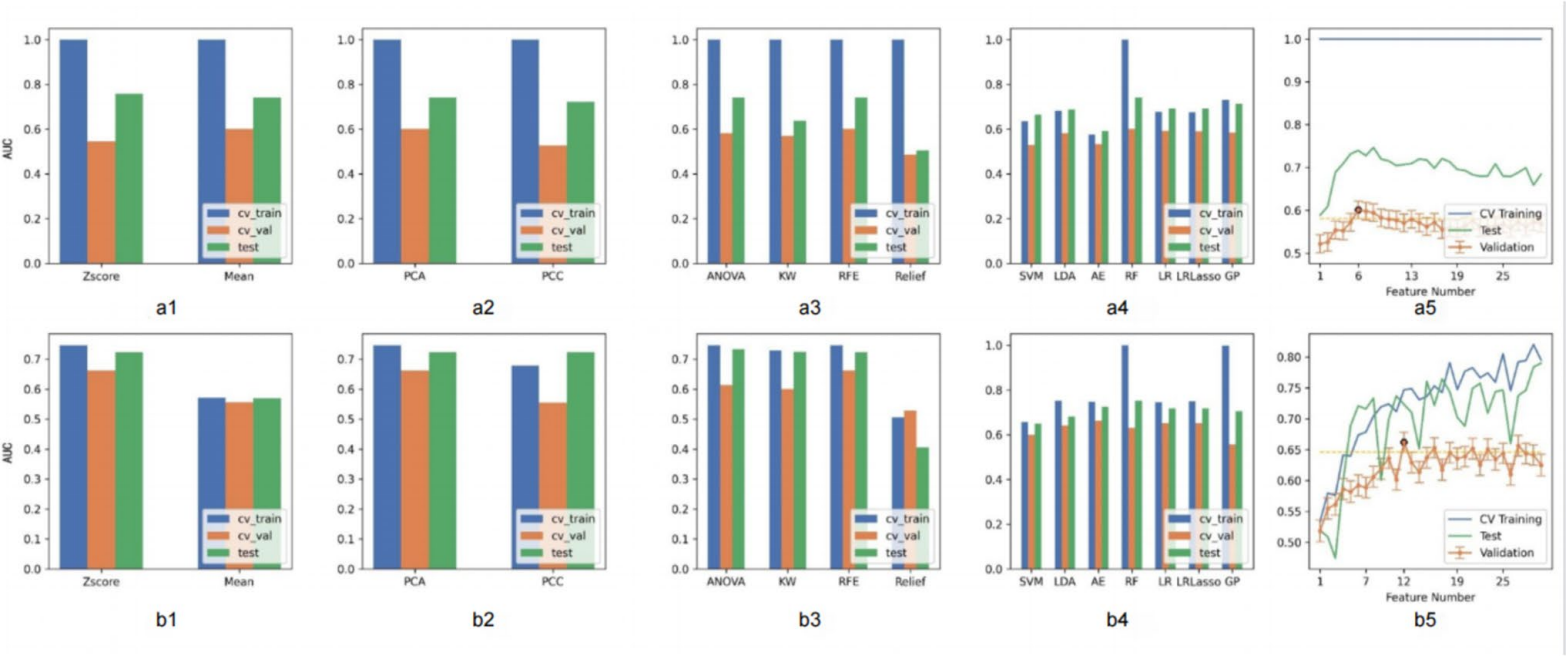
a, Radiomics model pre-NCRT; b, radiomics model post-NCRT. a1, b1, The impact of four normalization methods on the cross-training, cross-validation, and test sets, along with their respective AUCs; a2, b2, the impact of four feature dimension reduction methods on the cross-training, cross-validation, and test sets, along with their respective AUCs; a3, b3, the impact of four feature selection methods on the cross-training, cross-validation, and test sets, along with their respective AUCs; a4, b4, the impact of five model building methods on the cross-training, cross-validation, and test sets, along with their respective AUCs; a5, b5, the impact of 1–30 feature quantities on the cross-training, cross-validation, and test sets, along with their respective AUCs

As shown in Table 6, the optimal pre-treatment radiomics model was RF (Mean-PCA-RFE-6) and the optimal post-treatment model was AE (Zscore-PCA-RFE-12). The AUC relationships corresponding to the normalization mode, dimensionality reduction method, feature selection method, model selection, and number of selected features in the cross-training, cross-validation, and test sets of the ultrasound radiomics models before and after treatment are shown in Fig. 4.

**Table 6:** Different parameters in selected optimal Radiomics models pre- and post-NCRT

Parameters	Model	Normalization	Normalization	Feature selection	Number of selected features
Pre-NCRT	RF	Mean	PCA	RFE	6
Post-NCRT	AE	Z-score	PCA	RFE	12

*NCRT* neoadjuvant chemoradiotherapy, *RF* random forest, *AE* autoencoder, *PCA* principal component analysis, *RFE* recursive feature elimination

#### Feature selection and predictive efficiency of radiomics models

**Fig. 5:**
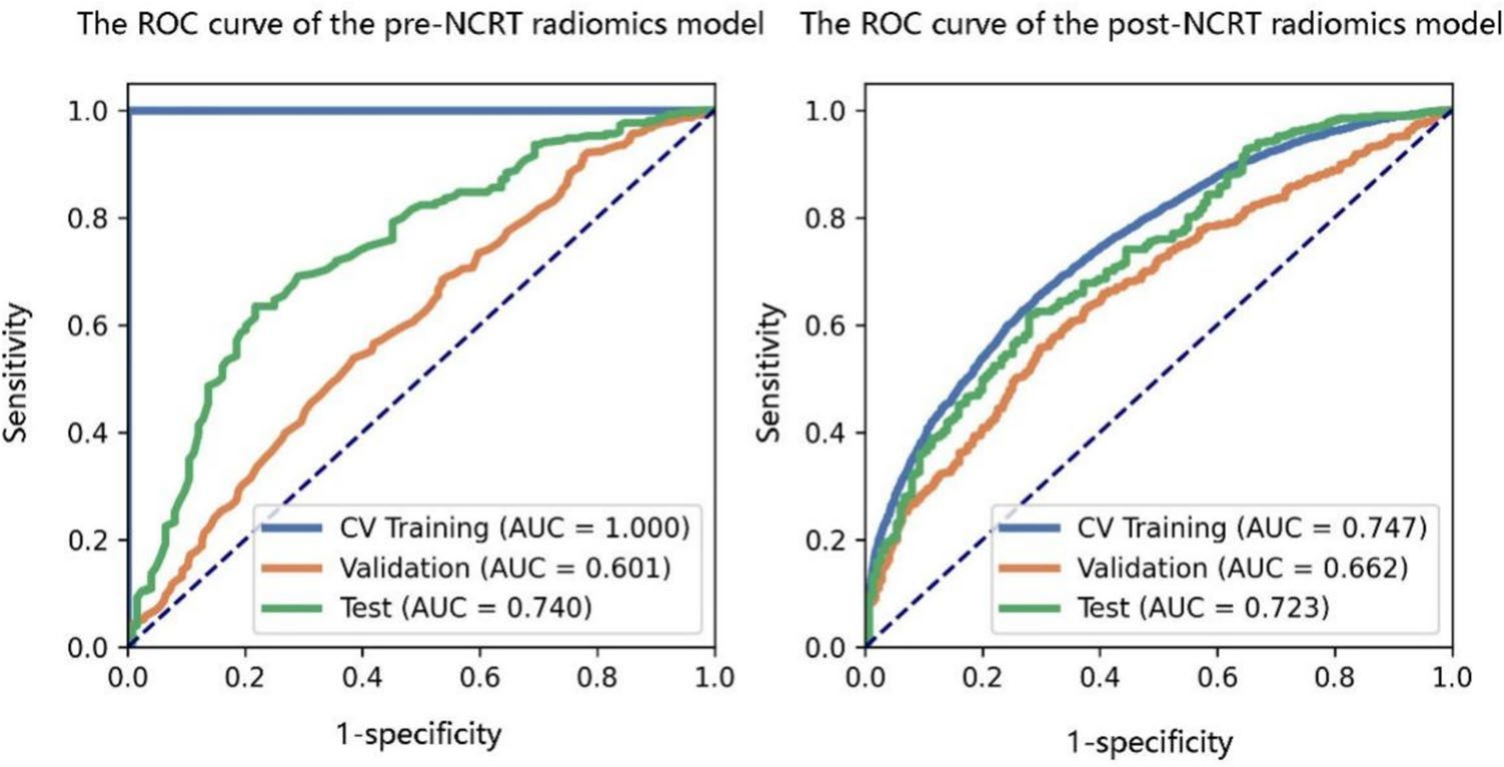
ROC and AUC values of imaging model RF in cross-training, cross-validation, and test sets before treatment (left); ROC and AUC values of imaging model AE in cross-training, cross-validation, and test sets after treatment (right)

PCA was the best dimensionality reduction method for both the pre- and post-NCRT ultrasound radiomics models. In PCA, the features in the new feature space are linear combinations of the original features. Therefore, their names are usually the numbers of principal components or projections rather than the names of the original features, and features with higher numbers have greater variance. The coefficients corresponding to all features before and after treatment were 1, indicating that all features contributed to the same degree (Table 7). The predictive efficiency of radiomics models based on pre- and post-NCRT 2-dimensional TRUS for distinguishing between good and poor NCRT responses was lower than expected (Table 8 and Fig. 5).

**Table 7:** Selected features and their coefficients in the models before and after NCRT

Before NCRT		After NCRT	
Mean_PCA_RFE_6_RF		Zscore_PCA_RFE_12_AE	
Feature	Coefficient	Feature	Coefficient
PCA_feature_2	1	PCA_feature_7	1
PCA_feature_10	1	PCA_feature_10	1
PCA_feature_17	1	PCA_feature_11	1
PCA_feature_21	1	PCA_feature_18	1
PCA_feature_29	1	PCA_feature_19	1
PCA_feature_45	1	PCA_feature_40	1
		PCA_feature_50	1
		PCA_feature_52	1
		PCA_feature_68	1
		PCA_feature_140	1
		PCA_feature_169	1
		PCA_feature_845	1

**Table 8:** Predictive performance of radiomics models pre- and post-NCRT in the cross-validation and testing sets

Stage	Model	Accuracy	AUC	AUC (95% CI)	Sensitivity	Specificity	PPV	NPV
Pre-NCRT	RF (Val)	0.564	0.601	[0.561–0.641]	0.538	0.615	0.737	0.400
	RF (Test)	0.670	0.740	[0.686–0.794]	0.615	0.782	0.850	0.503
Pre-NCRT	AE (Val)	0.596	0.662	[0.630–0.694]	0.557	0.704	0.838	0.366
	AE (Test)	0.602	0.723	[0.677–0.770]	0.548	0.748	0.857	0.377

*RF* random forest, *AE* autoencoder, *Val* cross-validation set, *Test* test set, *AUC* area under the curve

The potential reasons for this situation include the following: 1) Insufficient sample size. The number of ultrasound images used was still relatively small, which could have caused the model to learn the data’s noise and details during the training phase, rather than its intrinsic pattern and universality, potentially resulting in overfitting. 2) High model complexity. The employment of multiple feature extraction, dimensionality reduction, and model selection methods during the modeling process could have resulted in an excessively complex model that was more susceptible to fitting the training data’s noise, thereby diminishing its generalization ability. 3) Data imbalance. The significant disparity in the number of cases between GRG and PRG groups may have caused the model to disproportionately focus on the characteristics of the PRG group, thus compromising its prediction performance for the GRG group. In subsequent research, researchers will attempt to simplify the model, for example by reducing the number of features and lowering model complexity, to observe changes in model performance. If performance improves after simplification, it will suggest that the original model may have been overfitted. Researchers will also validate the model on external datasets and collect as many additional image data as possible to enhance the model’s generalization capability.

### Combined model of MPU-LR and radiomics

**Fig. 6:**
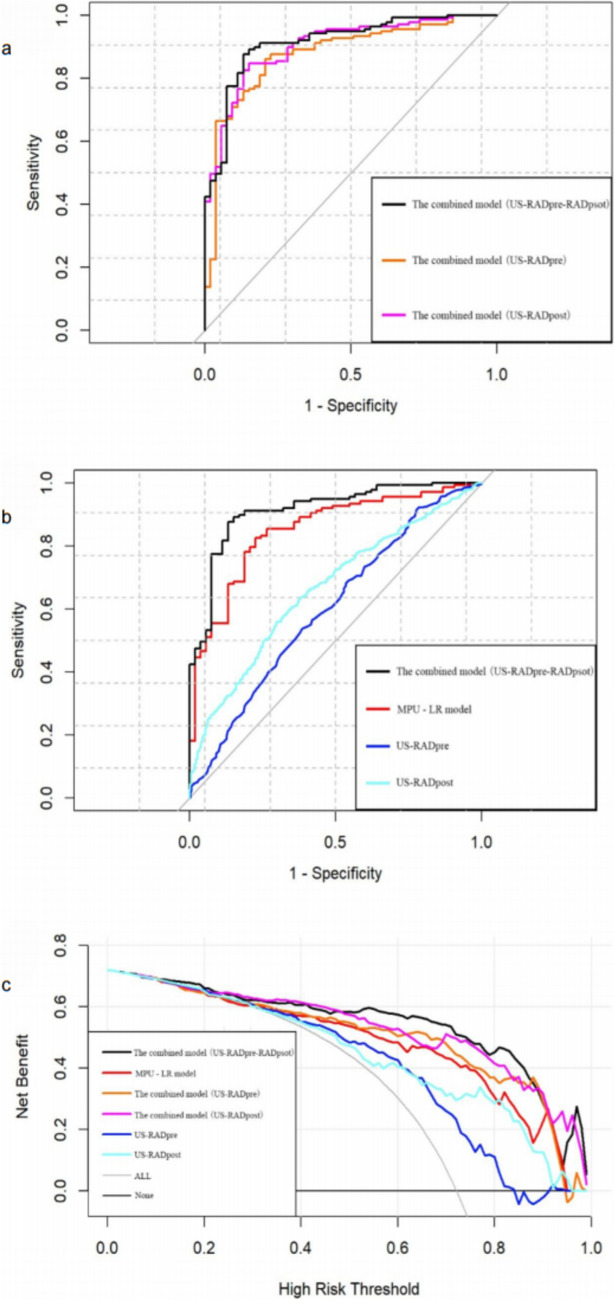
ROC curve and DCA curve of the combined model of multi-parameter ultrasound and radiomics. **a** ROC curves of three combined models. The AUC of US-RAD_pre_-RAD_post_ is 0.913, the AUC of US-RAD_pre_ is 0.877, and the AUC of US-RAD_post_ is 0.902. **b** Comparison of the combined model with MPU-LR model and the AUC of ultrasound radiomics. **c** Comparison of clinical net benefit between the combined, MPU-LR, and radiomics models

**Fig. 7:**
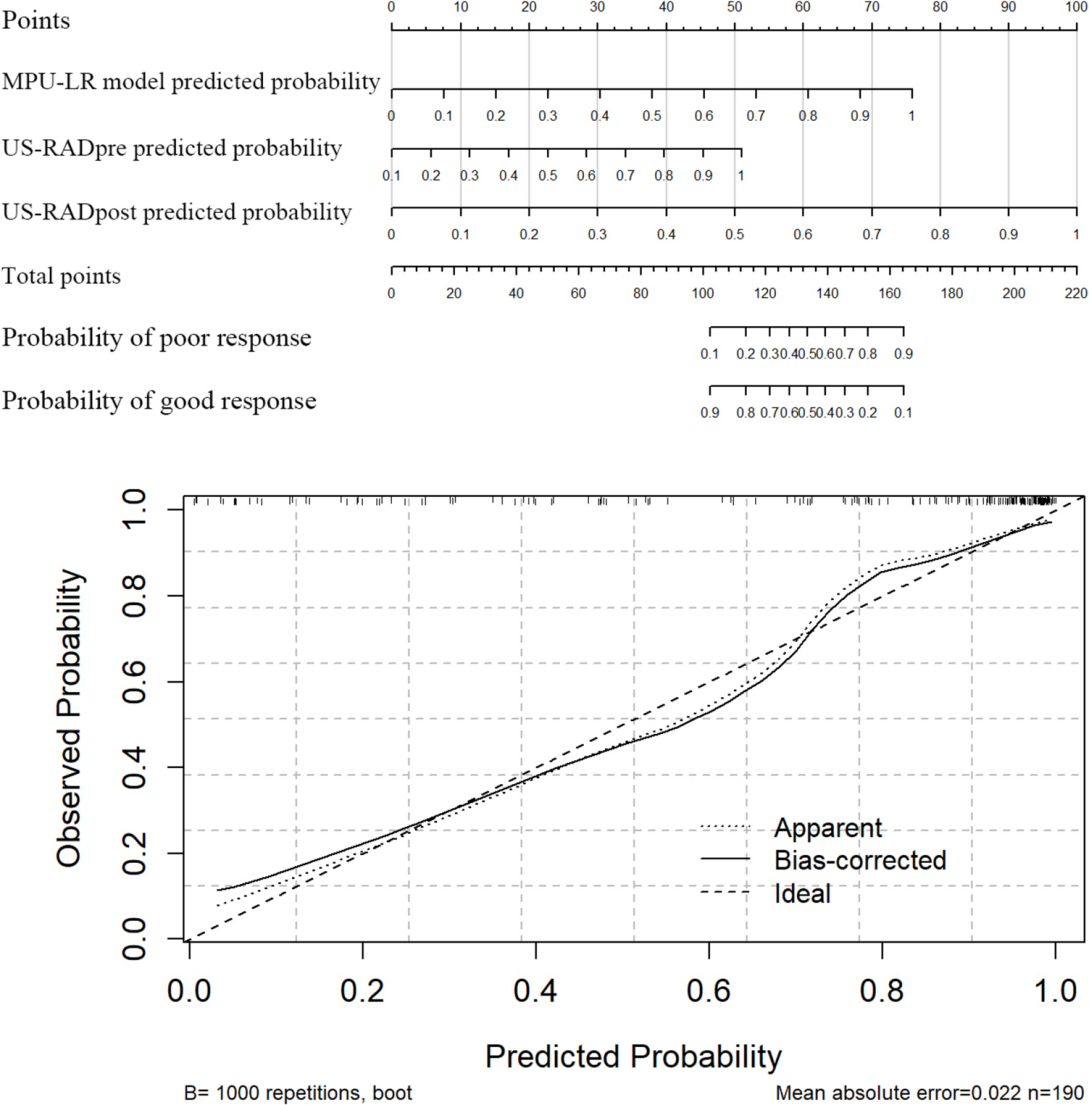
Nomogram of the combined model (US-RAD_pre_-RAD_post_) and its calibration curve

In this study, multi-parameter ultrasound, pre-treatment imaging (RAD_pre_), and post-treatment imaging (RAD_post_) were combined to predict TRG in patients with LARC. Among the three combined models constructed, the US-RAD_pre_-RAD_post_ combined model had the highest predictive efficacy, with an accuracy of 0.863 and an AUC of 0.913 (Fig. 6a). The Delong test (Table 9) and DCA curve showed that the AUC value and clinical net benefit of the comprehensive model were significantly higher than those of the MPU-LR model and the pre- and post-treatment radiomics models (US-RAD_pre_ and US-RAD_post_) (Fig. 6b and c). We developed a nomogram for the combined model, which could intuitively predict the outcome of patients through the prediction probability score of the MPU-LR model and the US-RAD_pre_ and US-RAD_post_ model; its calibration curve showed a good goodness of fit (Fig. 7).

**Table 9:** Delong test (US-RAD_pre_-RAD_post_ vs other models)

Model	AUC	Z/D value	*P* value
US-RAD_pre_-RAD_post_	0.913	-	-
US-RAD_pre_	0.877	2.3015	0.02136
US-RAD_post_	0.902	− 1.139	0.2547
MPU-LR	0.851	2.6954	0.00703
RAD_pre_	0.601	4.6929	< 0.001
RAD_post_	0.662	3.8174	< 0.001

*US-RAD*_*pre*_*-RAD*_*post*_ the combined model, *US-RAD*_*pre*_ the pre-treatment radiomics models, *US-RAD*_*post*_ the post-treatment radiomics model, *MPU-LR* the multi-parameter ultrasonic logistic regression model, *RAD*_*pre*_ pre-treatment imaging, *RAD*_*post*_ post-treatment imaging

## Discussion

The standard treatment for LARC comprises NCRT combined with TME, which can reduce tumor volume and staging, increasing the chances of sphincter preservation and improving local control and patient survival rates in rectal cancer. After NCRT, tumors undergo regressive changes, with TRG being a key indicator of NCRT effectiveness. If the TRG is accurately predicted preoperatively, it may be possible to avoid surgical intervention in patients who have achieved a complete pathological response, adopting a “watch-and-wait” strategy. This approach may reduce complications associated with surgery and the need for permanent stoma formation, allowing for personalized treatment plans and potentially improving the quality of life of patients [16]. Imaging is the primary method used to predict preoperative TRG. Patel et al. have previously suggested that mrTRG is a predictor of NCRT effectiveness, but the consistency between pathological TRG and mrTRG is debatable; subsequent studies have indicated that mrTRG cannot replace TRG [5]. Other imaging methods for predicting TRG based on tumor diameter and blood supply reduction have been explored, including diffusion-weighted imaging (DWI) and diffusion kurtosis imaging (DKI) in functional MRI [17–19]. However, the conclusions of these studies are not consistent and need to be verified by large-sample and multi-center studies. Conventional imaging methods have limited predictive value for TRG. In recent years, many studies have employed radiomics to predict TRG, among which MRI radiomics has shown higher efficacy in predicting TRG, offering a significant improvement over traditional imaging approaches. A study by Nie [20] et al. involving 48 patients with LARC undergoing NCRT compared radiomic texture analysis and conventional imaging volume measurement methods for TRG prediction and showed that the efficacy of the MRI-based imaging model was significantly higher than that of the conventional imaging method, with an AUC of 0.89. In a multi-center study with a larger sample size by Shaish et al. [21], MRI radiomics was used to predict TRG, with the constructed model showing an AUC of 0.80. However, the predictive capability of MRI radiomics for TRG spans a wide range, and there are differences in the radiomics methods utilized, with the accuracy and robustness of the models still requiring validation [22–24]. TRUS is cost-effective, with a low price tag, straightforward operation, and relatively low examination costs, making it more economical compared to bulkier imaging equipment like MRI [25]. During TRUS, patients are not required to be in a confined space or to lie flat on the examination bed for a prolonged period, which enhances patient comfort. TRUS clearly displays various layers of the rectal wall, providing detailed information about the tumor size, location, and depth of invasion. In this study, multiple TRUS measurement parameters and ultrasonic radiomics were combined to accurately predict TRG in patients with LARC.

The MPU-LR model was constructed using eight TRUS parameters that significantly differed between the groups: PSV_pre_, LD_post,_ TD_post_, CEUS-IG_post_, LD change rate, TD change rate, RI change rate, and CEUS-IG downgrade. The results showed that the prediction efficiency of the constructed MPU-LR model was significantly improved compared with that of a single ultrasonic parameter, with an accuracy of 0.816 and an AUC of 0.851 (95% CI: [0.792–0.909], *P* < 0.05), which was similar to the efficacy reported in previous studies [6, 26].

The FeAture Explorer software was used to explore various models of the extracted image radiomics features, which helped find parameters with the highest prediction efficiency for modeling. Additionally, a tenfold cross-validation was conducted to enhance the predictive performance of the model. A total of 3360 pre-treatment and post-treatment ultrasonic radiomics models were established. With the highest AUC value on the cross-validation set, the optimal pre-treatment radiomics model was selected as RF (Mean-PCA-RFE-6); accuracy was 0.563 and AUC was 0.601 in the cross-validation set. In the test set, the accuracy of the model was 0.670 and the AUC was 0.740. The post-treatment radiomics model selected was AE (Zscore-PCA-RFE-12), which had a prediction accuracy of 0.596 and an AUC of 0.662 in the cross-validation set and an accuracy of 0.596 and an AUC of 0.602 in the test set. The efficacy of the radiomics models constructed in this study was lower than that reported in previous studies [27]. Analyzing the relatively low efficacy of the radiomics models in this study, the underlying reasons may be multifaceted. Firstly, the TRUS images collected were sourced from two different ultrasound machines (Biosound MyLab Twice and BK Medical BK5000), and images from these two machines may exhibit heterogeneity. This heterogeneity could hinder the extraction of valuable features through the imaging radiomics workflow, leading to suboptimal performance of the imaging radiomics model. Despite the images having undergone normalization, the inter-image heterogeneity cannot be entirely eliminated. Secondly, it may be challenging to extract valuable features from grayscale ultrasound images, which could prevent the differentiation between GRG and PRG groups. Additionally, the sample size in this study was relatively small, and the number of extracted imaging radiomics features was limited. Only the original images and features derived from wavelet transformation were extracted, without attempting other transformations on the original images (such as LOG, filtering, Laplacian). To further analyze these issues, researchers plan to conduct a quality assessment of the ultrasound images, meticulously examining the potential impact of different ultrasound machines, probes, and operators on image quality. Concurrently, the efficacy of distinct image segmentation algorithms and noise reduction methodologies on model efficacy will be investigated. Furthermore, researchers will attempt different feature selection strategies and initiate a multi-center study to extensively collect patient data from different hospitals and regions to evaluate the model’s applicability and generalization ability in diverse populations.

In addition to the above reasons, there are other limitations of this study. This study was a retrospective study and did not consider the confounding effects caused by differences in the NCRT methods, which may be one of the reasons for the low efficiency of the radiomics model. Clinical indicators of patients, such as CEA and CA199, were not included in the study; the inclusion of these parameters in future analyses may improve the prediction efficiency of the model.

In this study, a combined model was constructed using the results of the MPU-LR and ultrasound radiomics models. Among various combinations, the combined model (US-RAD_pre_-RAD_post_) demonstrated the highest predictive performance, with an accuracy of 0.863 and an AUC of 0.913. Comparisons using the Delong test and DCA showed that the predictive performance and clinical net benefit of the combined model were significantly higher than those of the MPU-LR and radiomics models (*P* < 0.05). This combined model affords robust support for the precision treatment and prognostic evaluation of patients with LARC. Prior to surgery, by inputting TRUS imaging data of patients prior to NCRT, the model is capable of predicting TRG, thereby assisting clinicians in assessing patients’ responsiveness to NCRT and devising individualized treatment plans, such as prolonging NCRT duration, increasing chemotherapy dosage, or exploring more targeted therapeutic strategies. Postoperatively, utilizing TRUS imaging data to evaluate postoperative TRG facilitates a more precise assessment of NCRT efficacy, prediction of recurrence risk, and guidance for subsequent treatment adjustments. Moreover, regular TRUS and radiomics analyses can aid clinicians in promptly detecting tumor recurrence or metastasis, enabling early intervention and thus enhancing patient prognosis. Through multidisciplinary team collaboration, patient education, and long-term follow-up management, the TRUS and radiomics conjoint predictive model holds the promise of delivering more precise and effective treatment for LARC patients, thereby elevating their quality of life.

## Conclusion

The model constructed by combining multi-parameter TRUS and radiomics can accurately evaluate NCRT efficacy in patients with LARC. This may provide a reference for personalized treatment options and advanced precision medicine for patients with rectal cancer.

## Supplementary information

Below is the link to the electronic supplementary material.

ESM 1 (DOCX 13.0 KB)

## Data Availability

No datasets were generated or analysed during the current study.
