# Supplementary material for: Combined transrectal ultrasound and radiomics model for evaluating the therapeutic effects of neoadjuvant chemoradiotherapy in locally advanced rectal cancer
Source: Int J Colorectal Dis. 2025 Jan 7;40(1):7. doi: 10.1007/s00384-024-04792-8 (PMC11703880; doi:10.1007/s00384-024-04792-8)
Supplement: Supplementary file 1 — (DOCX 13.0 KB) [file 384_2024_4792_MOESM1_ESM.docx]

**Supplementary material 1 :**

**Feature selection methods**

In this study, the feature selection methods were used to select the features most relevant to the label data from among all available features. Feature selection involved multiple methods, including analysis of variance (ANOVA), the Kruskal-Wallis (KW) test, recursive feature elimination (RFE), and the relief algorithm.

**ANOVA (Analysis of Variance):**

ANOVA is a statistical method used to analyze the variance among multiple variables. It calculates the F-value for each feature relative to the label, which measures the feature's importance. Features are then ranked from highest to lowest based on their F-values, and the most relevant features to the label are selected. In this study, ANOVA was employed to assess whether there were significant differences in the means of each radiomics feature between different TRG grade groups. If significant differences were found, it indicated that the feature might be useful in differentiating between the various TRG grades, thus qualifying it as a potential effective biomarker.

**Kruskal-Wallis (KW) test:**

The KW test is a non-parametric statistical test designed for comparing three or more groups of data. It evaluates whether the samples are derived from the same distribution or if there are significant discrepancies between at least two of the samples. Specifically, it tests the null hypothesis that the populations from which the samples are drawn have identical distributions against the alternative hypothesis that at least one sample comes from a different distribution. In this study conducted, the KW test was used to determine whether there were significant differences in the medians of each radiomics feature among different TRG grade groups. If significant differences were identified, it suggested that the feature might be instrumental in distinguishing between different TRG grades, thereby qualifying it as a potential effective biomarker.

**Recursive Feature Elimination (RFE):**

The RFE is a feature selection method that involves repeatedly constructing a model (such as SVM or a regression model) and selecting the best (or worst) features based on their importance, often determined by the model's coefficients. The process involves the following steps:

1.Build a Model: Train a model using all available features.

2.Rank Features: Evaluate the importance of each feature, typically using coefficients or feature weights.

3.Eliminate Features: Remove the least important feature(s) from the dataset.

4.Repeat: Retrain the model on the remaining features and repeat the process until the desired number of features is reached.

The order in which features are eliminated corresponds to their ranking in terms of importance. This process helps to identify the most relevant features for the model.

**The Relief algorithm:**

The Relief algorithm evaluates feature relevance based on how well features distinguish between nearest neighbors of different classes. Here’s how the algorithm works:

1.Select a Sample: Randomly choose a sample R from the training set D.

2.Find Nearest Neighbors:

1)Identify the nearest neighbor H of the same class as R, called the Near Hit.

2)Identify the nearest neighbor m of a different class than R, called the Near Miss.

3.Update Feature Weights:

1)If the distance between R and H for a given feature is smaller than the distance between R and m for that feature, this indicates that the feature is effective in distinguishing between similar and different classes. Therefore, the weight of that feature is increased.

2)Conversely, if the distance between R and H is larger than the distance between R and m for the feature, it suggests that the feature negatively impacts class differentiation, and its weight is decreased.

4.Repeat: This process is repeated mmm times to update the feature weights.

5.Average Weights: After repeating the process, the average weight for each feature is calculated.

Features with higher weights are considered more effective for classification, while features with lower weights are less useful.
